# Study protocol: evaluation of the addictive potential of e-cigarettes (EVAPE): neurobiological, sociological, and epidemiological perspectives

**DOI:** 10.1186/s40359-021-00682-8

**Published:** 2021-11-18

**Authors:** Sabine Vollstädt-Klein, Nadja Grundinger, Tatiana Görig, Daria Szafran, Astrid Althaus, Ute Mons, Sven Schneider

**Affiliations:** 1 Department of Addictive Behavior and Addiction Medicine, Central Institute of Mental Health, Medical Faculty Mannheim, University of Heidelberg, PO Box 12 21 20, 68072 Mannheim, Germany; 2 Mannheim Center for Translational Neurosciences (MCTN), Medical Faculty of Mannheim, University of Heidelberg, Mannheim, Germany; 3 Mannheim Institute of Public Health, Social and Preventive Medicine (MIPH), Medical Faculty of Mannheim, University of Heidelberg, Mannheim, Germany; 4 Heart Center, Faculty of Medicine, University Hospital Cologne, University of Cologne, Cologne, Germany

**Keywords:** Electronic cigarettes, Addiction, Tobacco use disorder, Craving, Tolerance, fMRI, Focus groups, Longitudinal data

## Abstract

**Background:**

Tobacco use is the largest preventable cause of diseases and deaths; reducing tobacco intake is, therefore, an urgent public health goal. In recent years, e-cigarettes have been marketed as a 'healthier' alternative to tobacco smoking, whilst product features have evolved tremendously in the meantime. A lively scientific debate has developed regarding the potential benefits and risks of e-cigarettes although, surprisingly, there are few studies investigating the addictive potential of nicotine-containing e-cigarettes. The present work comprises three work packages investigating the addictive potential of e-cigarettes from different perspectives: (1) the neurobiological addictive potential of e-cigarettes; (2) the experience and perception of dependence symptoms among users of e-cigarettes in a social context; and (3) the epidemiological perspective regarding factors influencing the potential for dependence.

**Methods:**

Work package I: the neurobiological study will investigate the key elements of addiction in e-cigarettes compared to tobacco cigarettes using neurobiological and neuropsychological correlates associated with craving, incentive motivation, cue reactivity and attentional bias. Work package II: the sociological study part examines self-reports on the experience and perception of dependence symptoms in a social context, using focus group interviews and the analysis of posts in online discussion forums on e-cigarettes. Work package III: the epidemiological study part focuses on tolerance development and the role of psychosocial and product factors by analyzing longitudinal data from the International Tobacco Control Policy Evaluation Project (ITC).

**Discussion:**

The present study offers a chosen mix of three methodological approaches, thereby comprehensively examining core symptoms of positive and negative reinforcement in addiction. Whether e-cigarettes are as reinforcing and addictive as combustible tobacco cigarettes is an important public health issue with implications for prevention and treatment programs.

*Trial registration:* Work package I: Registered at clinicaltrials.gov/ct2/show/NCT04772014. Work package II: Registered at OSF Registries: https://osf.io/dxgya (2021, January 14).

## Background

Tobacco use causes more than 8 million deaths worldwide annually, making it the biggest preventable cause of disease [1]. Hence, reducing tobacco consumption and the associated health burden is an important goal. In 2006, e-cigarettes entered the market as alternatives to smoking tobacco cigarettes and the product characteristic have evolved tremendously since then. E-cigarettes can make nicotine available to users without exposing them to the harmful toxicants of tobacco smoke. Nevertheless, researchers have expressed different views on potential benefits and risks associated with e-cigarettes. Proponents see e-cigarettes as an innovative step in tobacco harm reduction, as switching from tobacco to e-cigarettes significantly reduces users’ exposure to the main toxicants of tobacco smoke [2–6] and may help smokers quit tobacco use [7]. Opponents on the other hand point to the lack of long-term data regarding potential health risks [8, 9], and fear that the marketing of e-cigarettes as lifestyle products leads to nicotine dependence and might be a gateway or a catalyst to tobacco use, especially among young non-smokers [10–14]. Although this 'gateway hypothesis' is highly controversial [15], research indicates that e-cigarettes—as compared to other nicotine replacement therapies (NRTs)—are mostly not limited to short-term use [16, 17]. Ex-smokers, who switch to e-cigarettes, often maintain their nicotine levels. While most e-cigarettes allow a gradual reduction of nicotine levels, longitudinal research findings indicates that a reduction in the concentration of nicotine may be accompanied by a higher consumption of liquid [18]. Such data suggests a maintenance of nicotine addiction that is initially acquired by tobacco use. It is however surprising that there are only few studies to date that examine the addictive potential of e-cigarettes containing nicotine.

Addictive behavior is determined by positive and negative reinforcement mechanisms. The rewarding potential of a substance includes its euphoric effect and the latency period until the effect occurs. The faster the drug enters the brain, the greater the euphoric effect [19, 20]. This positive reinforcement effect leads to an initial repetition of drug use. Neuroadaptive changes occur, which lead to a dysregulation of the neurochemical circuits and thus to withdrawal symptoms when the drug is discontinued [21]. The shorter the elimination half-life of a substance, the more severe the withdrawal symptoms [20]. To avoid withdrawal symptoms, the substance is consumed repeatedly and frequently, leading to increasing tolerance (e.g., higher dosage to achieve the same effect) [19]. Accordingly, in addition to positive reinforcement in the early stages of the addiction process, this negative reinforcement mechanisms are increasingly recruited as a source of motivation [21].

Tobacco dependence is primarily produced by the pharmacological effects of nicotine [22]. Cigarette smoke releases significant amounts of nicotine into the bloodstream, where it quickly reaches the brain and triggers the release of dopamine by stimulating nicotinic acetylcholine receptors [23]. Other characteristics and additives of tobacco smoke further enhance the addictive potential [24–27]. This causes the rapid positive reinforcement, making smoking of tobacco the most addictive form of nicotine administration [22, 28]. Nicotine uptake is significantly slower and lower with NRTs, which can explain the absence of the addiction-inducing 'kick' [29]. In most studies, e-cigarettes also showed lower nicotine absorption than tobacco cigarettes [30]. If they deliver nicotine less effectively, e-cigarettes might, thus, have less addictive properties. Nevertheless, depending on the device, liquid, and user behavior, it is possible to achieve equal or even higher plasma nicotine levels [30, 31].

However, nicotine is a necessary but not a sufficient component in the development of dependence [32]. Non-pharmacological motives for smoking include psychological, behavioral, sensorimotor and social manipulative factors [33, 34]. Thus, cigarette smoke is known to have very characteristic sensory effects on the respiratory tract that are perceived as pleasant and reduce the urge to smoke even more effectively than the direct pharmacological effect of nicotine [32, 35–37]. The combination of pleasant stimuli associated with smoking behavior and the drug itself act synergistically. Multisensory experiences of smoking (visual, tactile, auditory, olfactory, gustatory) quickly acquire the quality of a conditioned cue stimulus that can trigger the urge to smoke [38]. Therefore, craving can be induced by the substance itself, substance-associated stimuli or by emotional states such as stress [39, 40]. This is not necessarily associated with physical discomfort, but also includes preoccupation with thoughts of the drug. Expectations of positive outcomes from smoking (e.g., social interaction, stress coping, stop craving) as well as expectations of the negative consequences of quitting (e.g., physical withdrawal symptoms, weight gain) play a crucial role in addiction. Thus, it has been shown that 79% of interviewed ex-smokers are afraid of relapsing if they would stop using their e-cigarette [41]. Therefore, psychological dependence is also characterized by repeated drug use, but this is based less on tolerance development or physical withdrawal symptoms and more on classical and operant conditioning processes and craving [19]. With repeated consumption, the initial hedonic effects finally diminish, while consumption increasingly becomes habitual and eventually compulsive [42]. Cigarette smoking is such a compulsive behavioral pattern: rigid, automatic, and habitual actions that require little mental elaboration and are triggered by internal or external stimuli. E-cigarettes are the only tobacco-free nicotine delivery devices that closely resemble the smoking ritual of cigarette smoking: Hand-to-mouth movement, tactile action of puffing, inhalation and exhalation, the sensory stimulus in the airways, vapor production and social aspects such as smoking breaks. Therefore, e-cigarettes might be expected to produce the same psychological, behavioral, and social effects that can promote or maintain nicotine dependence.

Epidemiological data suggest that e-cigarettes may lead to dependence symptoms, such as craving, or e-cigarette use within 30 min of waking. In these studies, the severity of dependence, however, was significantly lower with e-cigarettes compared to tobacco cigarettes [43–45]. This is consistent with self-reporting by users, many of whom state that they are less dependent upon their e-cigarette than they were previously upon tobacco cigarettes [17, 46, 47]. Some experimental studies show that e-cigarettes, compared to other ‘high- and low-abuse liability’—nicotine products, have some risk of abuse that appears to be higher than for NRTs but lower compared to tobacco cigarettes [48–50]. Nevertheless, e-cigarette users show greater discounting for liquid compared to money, which was associated with more unsuccessful attempts to quit vaping [51].

Research to date shows that e-cigarettes have the potential for abuse liability and to maintain an existing nicotine dependence and lead to dependence symptoms. Whether e-cigarettes have a similar addictive potential as tobacco cigarettes has not yet been sufficiently clarified, especially since many studies were still conducted on old devices from earlier generations. Newer, more powerful devices can deliver nicotine more efficiently. It is still unclear what role dependence symptoms such as craving, tolerance and withdrawal symptoms play in e-cigarette use and how they develop.

This research hence comprises three work packages with the aim of investigating the addictive potential of e-cigarettes from three different perspectives, combining neurobiological, sociological, and epidemiological research methods and levels of observation. In particular, craving is examined as a correlate for reward potential and tolerance development as a correlate for punishment potential. By combining these complementary methodological approaches, the overall project aims to cover all relevant sub-constructs of the addictive potential of e-cigarettes.

## Work package I: neurobiological study part

Registered at clinicaltrials.gov/ct2/show/NCT04772014.

### Objectives

In this work package (WP), the focus is on the investigation of positive reinforcement mechanisms of e-cigarettes utilizing neurobiological and neuropsychological methods. One of the most discussed theories in this context is the incentive sensitization theory by Berridge and Robinson: Accordingly, mesolimbic sensitization occurs with repeated drug use, leading to a realignment of the reward and motivation system, resulting in the attribution of incentive salience of drug-associated stimuli, making them attractive and 'wanted' [52–54]. Thus, the substance and its stimuli are attributed a high reward value, which can be measured in terms of effort, time, money, or other goods one is willing to spend to acquire it. Some experimental studies on tobacco smokers [50, 55] and experienced dual users [56] show that tobacco cigarettes have a higher reward value than e-cigarettes. However, most participants smoked more frequently and for a much longer period of time, which is why the reward value for tobacco cigarettes could be more established. Thus, the reward value of cigarettes itself was found to differ between dependent and occasional smokers. Occasional smokers exert more physical effort to obtain money and showed increased reactivity of the mesocorticolimbic system (including ventral striatum) to stimuli that predicted a money reward compared with a cigarette reward. Dependent smokers, in contrast, exerted similar physical effort and showed equivalent anticipatory activity for both reward types [57]. Measuring brain activity in a heterogeneous e-cigarette consumer group during reward announcement and acquisition for tobacco cigarettes and e-cigarettes could provide additional information about their reward value.

In addition, numerous meta-analyses in tobacco smokers show that smoking-related cues elicit significantly greater craving in smokers than neutral cue stimuli. This is associated with distinctive neural activation patterns in, e.g., the striatum, amygdala, orbitofrontal cortex, anterior cingulate cortex, medial prefrontal cortex and insula [58–61]. There are few studies on cue reactivity with e-cigarettes and they deliver conflicting results. In a study with merely seven participants, Nichols and colleagues failed to detect e-cigarette cue-related activity in brain areas associated with cue reactivity; but in regions related to episodic memory retrieval and motor control [62]. Another study by Wall and colleagues examined 10 subjects using e-cigarettes during functional magnetic resonance imaging (fMRI) to visualize brain activity associated with active vaping. Activation clusters were seen in cortical regions, (e.g., the insula, amygdala, and the anterior cingulate gyrus) as well as in sub-cortical regions (e.g., in the thalamus and putamen). Relative deactivations associated with vaping were detected in parts of the ventral striatum [63]. One possible explanation may be the transition from goal‐directed to habitual behavior in e-cigarette use that has been associated with a dysfunction of fronto-striatal circuits and a shift from ventral to dorsal striatal responses [64].

Furthermore, cognitive processes play an essential role in reactivity to drug cues. Through mesolimbic sensitization drug stimuli automatically and involuntary become the focus of attention. Attentional bias has been consistently found in various substance use disorders [65] and also in smokers [66–69]. Studies have shown that smokers have an initial orientation to smoking-related cues [70] and maintain their gaze on smoking-related images longer than on control images [67, 71]. In fact, current tobacco smokers also have a longer dwell time on e-cigarette cues compared to neutral cues, which was associated with greater baseline craving [72]. However, to our best knowledge, there is no study investigating attentional bias for tobacco cigarette and e-cigarette stimuli in e-cigarette users.

In sum, the neuropsychological and neurobiological mechanisms of cigarette dependence have been relatively well studied. The extent to which this can be applied to e-cigarettes has not yet been adequately elucidated. Therefore, the neurobiological study will investigate the key elements of addiction in e-cigarettes compared to tobacco cigarettes using neurobiological and neuropsychological correlates associated with craving, incentive motivation, cue reactivity and attentional bias. We hypothesize that (1) participants who mainly use e-cigarettes work harder for e-cigarettes and show increased activation in the ventral striatum in the anticipation phase for e-cigarettes compared to tobacco cigarettes; (2) e-cigarette users show increased cue reactivity (compared to nicotine naïve users) in response to e-cigarette stimuli compared to neutral stimuli. Moreover, dual users will show activations in the same neural networks for tobacco cigarette and e-cigarette stimuli; (3) e-cigarette users (compared to nicotine naïve and dual users) show an increased attentional bias towards e-cigarette cues, which correlates positively with e-cigarette use. Dual users' attentional bias towards smoking cues, on the other hand, correlates positively with tobacco cigarette use.

### Methods

#### Study sample

We intend to include 70 e-cigarette users (daily e-cigarette use, additional smoking of tobacco cigarettes is not an exclusion criterion) and 30 nicotine naïves (lifetime consumption of less than 20 e-cigarettes or tobacco cigarettes) aged 18–65 years. Exclusion criteria for both groups are contraindications for an MRI examination, severe internal, neurological, and psychiatric comorbidities, pharmacotherapy with psychoactive substances within the past 14 days, current substance abuse (THC, amphetamine, opiates, benzodiazepines, barbiturates, and cocaine) and axis I disorders according to ICD-10 and DSM-5 (except tobacco use disorder and specific phobias).

#### Power calculation

Sample size was estimated with an assumed effect size of ρ = 0.3 using G*Power software tool version 3 [73]. In this case, n = 64 smokers would be sufficient to detect a correlative relationship at *p* < 0.05 with a power of 80%. Since dropouts due to artifacts or lack of compliance are to be expected, a total of 70 subjects will be examined. The number of cases in the healthy control group (n = 30) is also sufficient to detect group differences between smokers and nicotine naïve subjects (effect size d = 0.6; 80% power, *p* < 0.05).

#### Study design

**Fig. 1:**
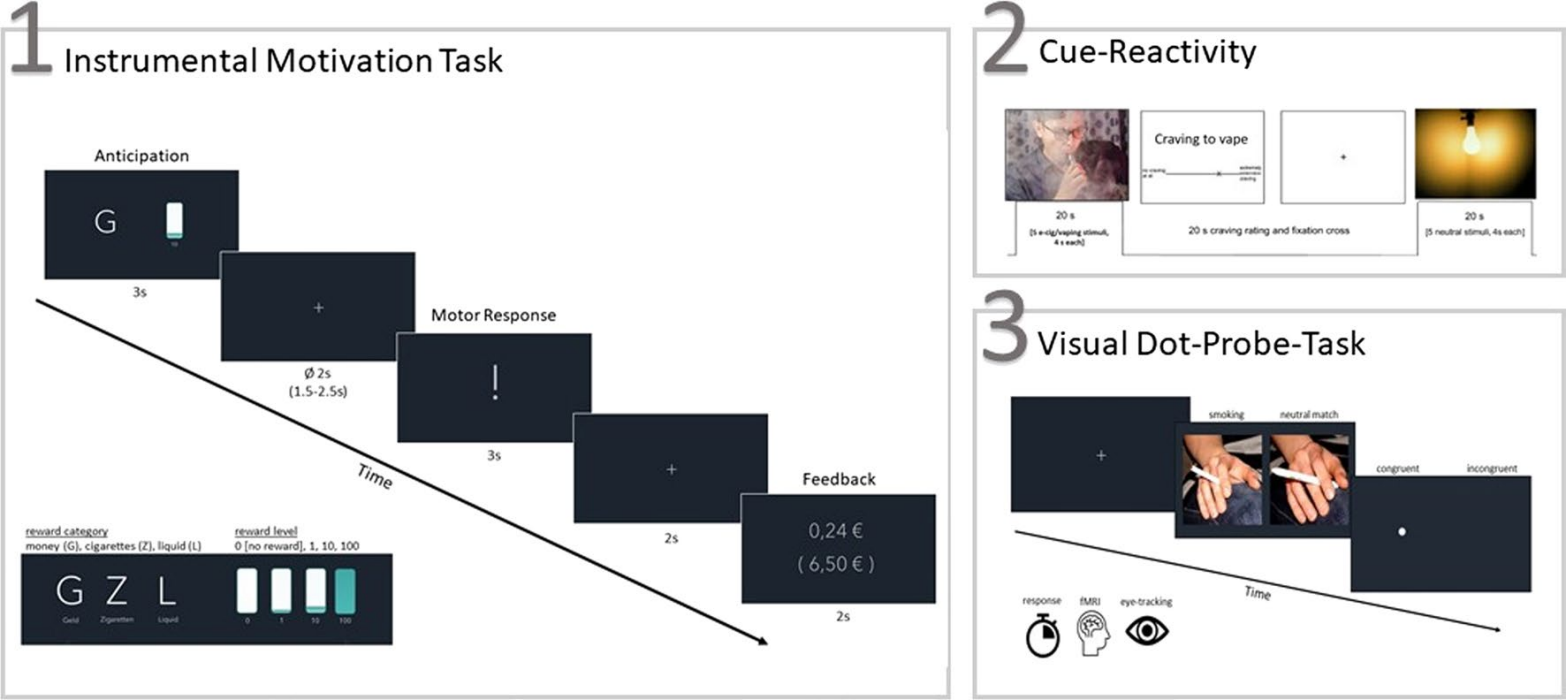
Experimental tasks used during fMRI. *Note.* (**1**) Instrumental motivation task (adapted from [57]); (**2**) Cue-reactivity paradigm (adapted from [74]); (**3**) visual dot-probe task (adapted from [69])

Inclusion and exclusion criteria are checked in advance in a telephone interview. The subjects are comprehensively informed about the objectives and the procedure of the planned examinations. On the examination day, smoking status is checked by measuring carbon monoxide levels in exhaled air and by taking a saliva sample to determine cotinine levels. Sociodemographic data are collected. Drug urine screening is performed, as well as a pregnancy test for women. This is followed by diagnostic interviews and the recording of smoking and vaping behavior, the severity of dependence symptoms, craving, and expected consequences for the use of tobacco cigarettes and e-cigarettes, as well as withdrawal symptoms using standardized questionnaires. Psychiatric and neurological, as well as somatic pre-existing conditions are recorded, as is the subject's current medication. In a neuropsychological assessment, subjects complete an Implicit Association Task, Delay Discounting Task, and Iowa Gambling Task. The fMRI examination is performed with a 3 T whole-body tomograph (Siemens Healthineers, Erlangen, Germany) including resting-state, MPRAGE and three different tasks: (1) To measure the reward value of e-cigarettes, we use an instrumental motivation task (adapted from [57]). Thereby, we examine brain activity to reward-predicting stimuli (cigarette, liquid, and money) and the subsequent instrumental response to obtain the reward. Physical effort (pressing a button) is thus used as a measure of motivation. (2) In a cue reactivity paradigm (adapted from [74]) participants’ physiological and neural responses, as well as self-reported craving, are examined while viewing images of e-cigarettes and tobacco cigarettes compared to neutral pictures. (3) Attentional bias for e-cigarette and tobacco cigarette cues and its neural correlates are tested using a visual dot-probe task (adapted from [69]). One problem is that the reaction time-based index only provides a 'snapshot' of attention, which can be overcome by directly measuring participants' eye movements during the task. To improve reliability, this task is, therefore, combined with eye tracking (see also [75, 76]). For a comprehensive list of questionnaires and tasks used, please see Table 1. For a graphical representation of the paradigms used during fMRI, please see Fig. 1.

**Table 1:** Measurements implemented in WP I

Initial information	Sociodemographic data
	Structured Clinical Interview for DSM-5 [SCID-I; 77]
	Preexisting conditions and medication
	MRI suitability
	Drug screening
	Pregnancy test (for women)
Nicotine consumption	CO-measurement
	Saliva sample (cotinine)
	E-cigarette use (device, liquid, consumption pattern)
	Smoking and vaping history
	Form 90 interview for smoking and vaping [adapted from 78]
	Fagerström Test of Cigarette Dependence [FTCD; 79]
	Penn State Electronic Cigarette Dependence Index [PS-ECDI; 17]
	Craving-Automatized-Scale for Cigarette Smoking [CAS-CS; 80]
	Craving-Automatized-Scale for Vaping [CAS-V; 80]
	Questionnaire of Smoking Urges [QSU; 81]
	Questionnaire of Vaping Craving [QVC; 82]
	Smoking Consequences Questionnaire [BSCQ-A; 83]
	Vaping Consequences Questionnaire [VCQ; adapted from 82]
	Minnesota Tobacco Withdrawal Scale [MTWS-R; 84]
Emotional state	Perceived Stress Scale [PSS; 85]
	Positive And Negative Affect Schedule [PANAS; 86]
	Barratt-Impulsiveness-Scale [BIS-11; 87]
Neuropsychological assessment	Implicit Association Task [88]
	Kirby Delay Discounting Task [89]
	Iowa Gambling Task [90]
fMRI examination	Resting-state
	MOTTA-task [57]
	Cue-reactivity task [74]
	Visual dot-probe-task combined with eye-tracking [69]
	MPRAGE

## Work package II: sociological study part

Registered at OSF Registries: https://osf.io/dxgya.

### Objectives

In this part of the study program, the question is whether the reward potential (e.g., craving) and the punishment potential (e.g., tolerance development) are actually subjectively perceived by e-cigarette users. Therefore, the aim of the sociological study part (WP II) is to investigate whether aspects of addiction defined in the current DSM-5 are also reported by the users themselves. To this end, two different qualitative approaches will be combined.

### Methods

#### Focus group interviews

One approach within the sociological study part (WP II) is to conduct four focus group interviews, each with 9–10 e-cigarette users. The planned focus group interviews aim to capture potential experiences of craving and tolerance development. An open-ended guide will be developed for conducting the focus group interviews. Inclusion criteria for participants will be: (1) age ≥ 18 years, (2) sufficient understanding of the German language, and (3) daily e-cigarette use. Dual use of tobacco and e-cigarettes will be set as a specific exclusion criterion for participation in this study part to exclude dependence symptoms resulting from the tobacco cigarette use. The composition of the focus groups will be as heterogeneous as possible [91] in order to generate a wide variety of opinions and to discuss as many different experiences as possible.

Prior to the focus group interview, each participant will be personally informed about the objectives of the study and data protection procedures. A written consent will be obtained from each participant. After completion of each focus group (max. 2 h), all participants will receive information on the current state of research on health risks and dependence potential of e-cigarettes. In addition, participants will receive an expense allowance of 50€ and the opportunity to receive information about the study results at a later date. All focus group discussions will be audiotaped, transcribed verbatim, and analyzed using qualitative content analysis [92].

#### Online forums

In addition to the focus group interviews, an analysis of posts in online discussion forums on e-cigarettes will be conducted. Online forums represent anonymous places of exchange for users [93] and an opportunity to share ideas about problematic or taboo topics without fear of stigmatization [94]. We hypothesize that the internet, and in particular anonymous online forums, are among the few places where e-cigarette users report possible dependence symptoms without feeling shame. Therefore, they provide a venue to gain health- and dependence-related experiences without the social-desirability bias [95]. Examining posts in online forums complements focus group interviews in an innovative and useful way, as shame-related experiences may be reported more detailed than in focus group interviews.

A three-step procedure will be used to collect the data for this study part. In the first step, the relevant online forums will be identified via Google search using different combinations with various spellings of the German words “e-cigarette” and “(online) forum”. Following inclusion criteria will be applied to select relevant online forums: (1) e-cigarettes as the main topic of the forum, (2) the forum is in German language; (3) the forum is publicly accessible (i.e., no registration is required to read the users contributions, (4) the forum was active over the previous 4 weeks, (5) at least 500,000 posts, (6) at least 5000 forum members, (7) search function within the forum, (8) no affiliation with tobacco industry, (9) public disclaimer in terms and conditions. In the second step, the identified forums will be searched for previously defined keywords describing dependence criteria derived from current DSM-5 covering the reward potential (e.g., craving) and the punishment potential (e.g., tolerance development) as accurate as possible. In the third step, the identified user contributions will be analyzed using qualitative content analysis in regard to the reported dependence symptoms.

As suggested in a previous discussion about ethics of using of online data [96], formal ethical clearance is not necessary for analyses of such kind of posts in online discussion forums. We will use data that is publicly accessible at the time of data collection, so that forum members can be assumed to be aware of the public availability of their posts. Nicknames of users will not be included in data analyses, and no further information on individuals is available in the forums. The team members will not participate actively in any discussions in the forums.

## Work package III: epidemiological study part

### Objectives

The aim of WP III is to quantify dependence symptoms, in particular the development of tolerance, in e-cigarette users and to investigate associations with user and product factors within the framework of a secondary data analysis of a representative large-scale longitudinal study of tobacco and e-cigarette use. In particular, we will investigate how dependence symptoms develop over time. For this purpose, transitions to e-cigarette use or from tobacco cigarette use will be investigated. Associations of such transitions with individual factors (e.g., age, gender, socioeconomic factors) and attitudes and perceptions (e.g., perceived dependence and harm potential of tobacco and e-cigarettes, perceived societal norms regarding tobacco and e-cigarette use) will be studied. In addition, because most e-cigarette users were previously long-time smokers of conventional tobacco cigarettes or continued to smoke tobacco cigarettes, the comparison of the perceived addictive potential of both products is of interest.

### Methods

WP III mainly includes a secondary data analysis of already collected and available longitudinal data from the International Tobacco Control Policy Evaluation Project (ITC)—a multinational consortium comprising longitudinal surveys on representative samples of smokers using largely standardized survey instruments and methods [97]. Conceptually, the survey instruments and models of the ITC cigarette project are based on psychosocial behavioral theories [98]. For the planned analyses, two survey waves are used, which were collected in six European countries (Germany, Greece, Poland, Romania, Spain, Hungary) within the EU-funded project EUREST-PLUS [99]. The baseline survey took place from June to September 2016 and included approximately 1000 smokers per country (total: N = 6011). Participants were recruited using multistage cluster sampling, geographically stratified by Nomenclature of Territorial Units for Statistics-Region (NUTS) region. A random walk procedure was used to randomly select addresses of households from 100 clusters in each country. Households were eligible if at least one smoker (> 100 cigarettes smoked in lifetime and at least monthly cigarette consumption) lived there. A maximum of one male and one female smoker from each selected household were randomly selected for a computer-assisted interview [100]. The second wave of the survey took place between February and May 2018, during which participants from the first wave of the survey were interviewed a second time. Overall, 54% of participants from the first wave participated a second time. For participants who refused to participate a second time or could not be reached (so-called panel mortality), replacement participants were recruited analogous to the initial sample selection to enable cross-sectional analyses in addition to longitudinal analyses [101]. This database is supplemented by two further surveys of the ITC cigarette Europe project (the Netherlands: approx. N = 2000 smokers, and England: approx. N = 4300 smokers, former smokers, and vapers), which were not collected within the scope of the EUREST-PLUS project, but which have good comparability given the use of the ITC cigarette sampling design and data collection methods across all involved countries [101]. All study participants provided informed consent and all study procedures and material were approved by the ethics research committee at the University of Waterloo (Ontario, Canada), and local ethics committees in all countries.

Of relevance for this work package are dependence symptoms, which were recorded with identical question wording at both survey time points and separately for tobacco cigarettes and e-cigarettes, depending on which products are being used. Pertinent measures include, in particular, self-reports (e.g., time of first use of cigarettes or e-cigarettes after getting up in the morning, failed attempts at abstinence, need for daily functioning) and self-assessments (e.g., of the degree of dependence). Furthermore, measures of perceived addictiveness of products are available. Tolerance development can be mapped longitudinally via detailed recording of dose for both tobacco cigarettes and e-cigarettes.

Measures of interest and associations with individual and product factors will be studied cross-sectionally using regression models. To examine the course of dependence symptoms over time based on longitudinal data, generalized linear models are the method of choice to account for intra-individual correlation. In order to investigate to what extent trajectories or transitions are influenced by individual and product characteristics, these are introduced into the models as influencing factors (i.e., modeled as interaction terms with the time factor).

## Discussion

The addictive potential of tobacco cigarettes is undisputed. E-cigarettes are very similar to tobacco cigarettes in their nicotine delivery and smoking behavior: Hand-to-mouth movement, tactile action of puffing, inhalation and exhalation, sensory stimulation in the airways, nicotine uptake via the pulmonary route, vapor production and social aspects such as smoking breaks. Therefore, e-cigarettes could produce the same pharmacological, psychological, behavioral, and social effects that can promote or maintain nicotine dependence. However, there are few studies on the addictive potential of e-cigarettes containing nicotine—with conflicting results. With the present project, we aim to close this gap by investigating the addictive potential of e-cigarettes from three perspectives, combining different research methods and levels of observation:

1. The neurobiological study part focuses on the positive reinforcement mechanisms of e-cigarettes using neurological and neuropsychological research methods. On the neurobiological level, the reward value of e-cigarettes and craving will be investigated in an experimental approach through presentation of conditioned stimuli and measurement of motivational and attentional processes. The aim is to test the assumption that chronic use of e-cigarettes leads to similar conditioning processes and motivational aspects as with traditional tobacco cigarettes.
2. The sociological study part uses a qualitative approach to investigate the extent to which e-cigarette users actually experience and report craving and tolerance development. This involves a qualitative description of the typical experience and perception of dependence symptoms in a social context. The self-reports of users cover psychological, physiological, and behavioral aspects of dependence disorders.
3. The epidemiological study part examines the factors influencing the potential for dependence and the development of dependence symptoms in a longitudinal study. The focus is on the development of tolerance and the role played by psychosocial and product factors with regard to transitions into and out of e-cigarette use. Using readily available quantitative longitudinal data, the development of tolerance among e-cigarette users will be quantified and associations with user and product factors examined.

During the conduct of this project, we will have to deal with certain limitations. Most e-cigarette users are former or current smokers. Here, the dependence symptoms or the dependent behavior could reflect the transfer of nicotine dependence from the previous use of combustible tobacco. Thus, it cannot be clarified whether the use of e-cigarettes alone actually leads to the development of addiction. Ideally, an evaluation of the addictive potential would be done with a group of individuals using nicotine-containing e-cigarettes but who have never used tobacco products. However, the prevalence of exclusive e-cigarette users who have never smoked cigarettes in their life is very low. Furthermore, previous or concurrent cigarette use also plays a role in the examination of reward effects such as craving and incentive motivation. Thus, most users have a longer smoking history and may have a greater sensitivity to tobacco cigarette and smoking stimuli. Therefore, we collect extensive smoking variables (age of smoking initiation, duration of smoking, exposure to tobacco cigarettes measured by pack years, severity of dependence on tobacco cigarette) to statistically account for these potential confounders. From a methodological point of view, the heterogeneous product group of e-cigarettes and different liquids and nicotine concentrations must also be taken into account. This can make it difficult to analyze and compare the data collected. Therefore, extensive information about e-cigarette consumption is collected (e-cigarette device, coil model, nicotine concentration, flavor etc.). Nevertheless, e-cigarettes differ not only in their characteristics, nicotine delivery and consumption patterns, but also in their design, which makes it difficult to select suitable stimuli. This is problematic given that personalized, familiar stimuli can best trigger craving and attention biases [75].

The strength of the present study is the chosen mix of three methodological approaches, whereby core symptoms of positive and negative reinforcement in addiction are investigated comprehensively. The reward effects of e-cigarette and craving can be experimentally validated at the individual level, while longitudinal designs are the method of choice for measuring tolerance development as a correlate of the punishment potential. The simultaneous individual occurrence of both phenomena can furthermore be investigated through qualitative analysis of self-reports.

Whether e-cigarettes are as reinforcing and addictive as combustible tobacco cigarettes is an important public health question with implications for prevention and treatment programs. In particular, the development of tolerance towards e-cigarettes is relevant from a public health perspective, as health risks are usually higher with increasing consumption. In contrast, a systematic assessment of the reward value of e-cigarettes, especially in comparison to tobacco cigarettes, plays an important role for therapy offers. The results will provide important insights into the motivational properties of e-cigarettes and could expand our understanding of whether and to what extent e-cigarettes can be used in smoking cessation treatments.

## Data Availability

Not applicable.

## Abbreviations

fMRI: Functional magnetic resonance imaging
ITC: International tobacco control policy evaluation project
NRT: Nicotine replacement therapy
WP: Work package
